# Mapping a path forward: addressing disease burden, pathways and solutions in ANCA-associated vasculitis

**DOI:** 10.1093/rheumatology/keaf517

**Published:** 2025-10-06

**Authors:** Bernhard Hellmich

**Affiliations:** Klinik für Innere Medizin, Rheumatologie, Pneumologie, Nephrologie und Diabetologie, Medius KLINIKEN Kirchheim-Teck & Nürtingen, Akademisches Lehrkrankenhaus der Universität Tübingen, Kirchheim unter Teck, Germany

**Keywords:** Anti-neutrophil cytoplasmic antibody, ANCA-associated vasculitis, AAV, induction therapy, maintenance therapy, treatment guidelines, avacopan

## Abstract

Anti-neutrophil cytoplasmic antibody (ANCA)-associated vasculitis (AAV) is a frequently relapsing systemic autoimmune disorder characterized by inflammation and destruction of small- to medium-sized blood vessels resulting in potentially life-threatening organ damage. Of the three AAV subtypes, granulomatosis with polyangiitis (GPA) and microscopic polyangiitis (MPA) are the most common. The aims of treatment are to rapidly control active disease with induction therapy [typically rituximab (RTX) (the new standard-of-care) or cyclophosphamide alongside glucocorticoids (GC) and avacopan], followed by less aggressive maintenance strategies to reduce the risk of relapse. International and national guidelines for the treatment of GPA/MPA are generally aligned, with all guidelines highlighting a need to reduce treatment-related adverse events through rapid GC tapering and the use of GC-sparing avacopan treatment. Guidelines will continue to evolve as ongoing studies provide new insights into alternative (GC-sparing) treatment options and optimal RTX-based treatment regimens.

Rheumatology key messages

- International and national guidelines for the treatment of AAV are generally aligned.
- Rituximab is recommended as the new standard-of-care for patients receiving induction and maintenance therapies.
- All guidelines highlight a need to reduce glucocorticoid exposure via rapid tapering and adjunctive treatment with avacopan.

## Introduction

Anti-neutrophil cytoplasmic antibody- (ANCA-) associated vasculitis (AAV) is a group of three genetically distinct autoimmune conditions characterized by inflammation and necrosis of small- and medium-sized blood vessels throughout the body. The articles in this supplement discuss the most common types of AAV: granulomatosis with polyangiitis (GPA) and microscopic polyangiitis (MPA) [1–3]. Eosinophilic granulomatosis with polyangiitis (EGPA), another form of AAV that is associated with asthma eosinophilia, is beyond the scope of this supplement.

In Europe, GPA is typically associated with polymorphisms in the genes encoding HLA-DP, SERPINA-1 and PRTN-3 leading to the production of ANCA directed against proteinase 3 (PR3), whereas MPA is more commonly associated with polymorphisms in the HLA-DQ gene and ANCA against myeloperoxidase (MPO) [1, 3, 4]. Vasculitis and chronic inflammation are key features of both GPA and MPA [1]. In addition, patients with GPA often have granulomatous inflammation, especially in the lungs and the ears, nose and throat (ENT) [1]. Early AAV is typically associated with a range of symptoms associated with chronic inflammation, including fatigue, weight loss, fever, myalgia, polyarthralgia [2] and (in patients with GPA), ENT-related manifestations [1]. As the disease progresses, patients may develop impairments affecting the kidney, lungs, skin, mucous membrane/eyes, nervous system and/or heart [2]. The risk of developing irreversible and potentially fatal organ damage increases the longer AAV remains undiagnosed and untreated. Consequently, the aims of treatment are to rapidly control active disease using early and intensive induction therapy, followed by less aggressive maintenance therapies to reduce the risk of AAV relapse [5].

The increasing number of AAV clinical trials over recent years has led to revised AAV treatment guidelines from the European Alliance for the Association of Rheumatologists (EULAR) (2024) [5], KDIGO (2024) [6] and numerous country-specific recommendations [7–13], the last of which was published by the British Society of Rheumatology (BSR) in 2025 [14].

## Induction therapy

**Figure 1.**
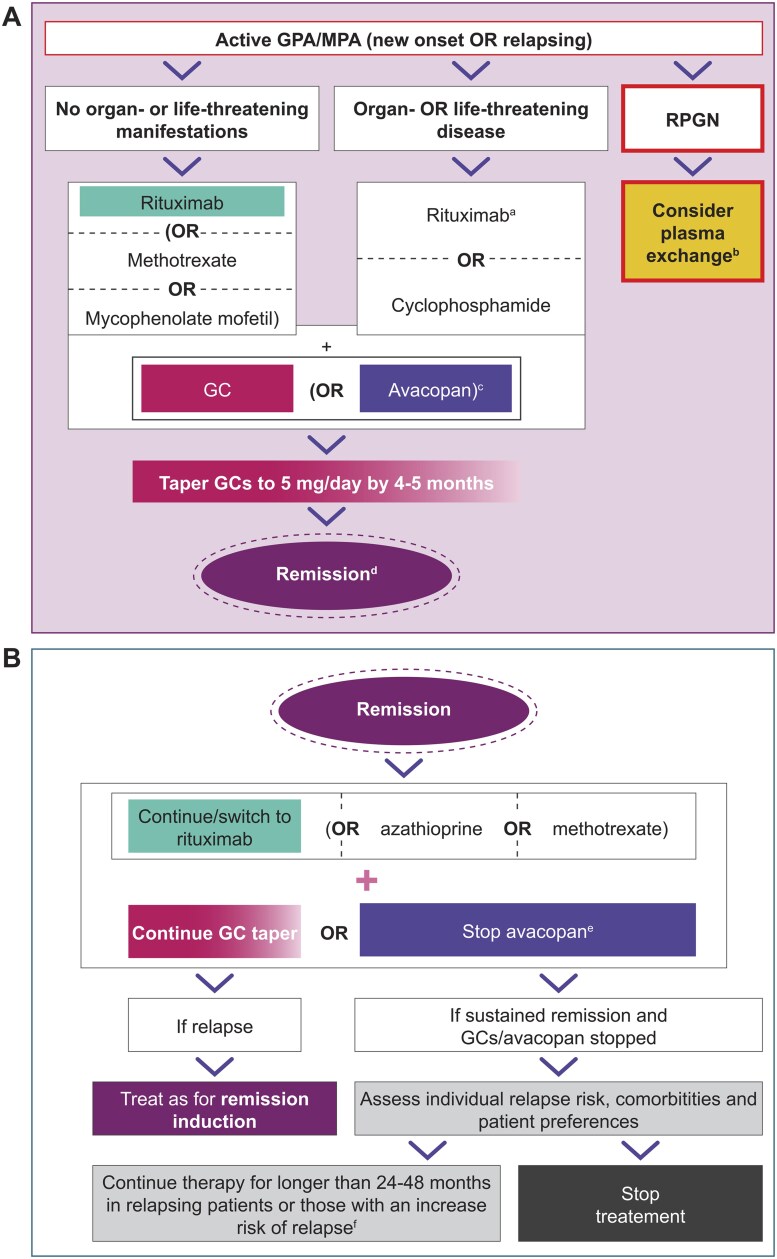
EULAR-recommended induction therapy (**A**) and maintenance therapy (**B**) for the treatment of GPA/MPA [5]. Diagrams adapted from Hellmich B, et al. [5]. ^a^Rituximab is preferred in patients with relapsing disease. ^b^In patients with serum creatinine >300 μmol/L due to active glomerulonephritis. ^c^As part of a strategy to substantially reduce GC exposure. ^d^If remission is not achieved, consult an expert centre; if remission is achieved, proceed to maintenance. ^e^Stop avacopan after 6–12 months (longer-term use cannot be recommended due to lack of data beyond 1 year). ^f^Longer treatment durations should be balanced against patient preferences and the risks of immunosuppression. EULAR, European Alliance of Associations for Rheumatology; GC, glucocorticoid; GPA, granulomatosis with polyangiitis; MPA, microscopic polyangiitis; RPGN, rapidly progressing glomerulonephritis

EULAR guidelines recommend treating patients with active, organ- or life-threatening GPA or MPA [i.e. those with glomerulonephritis (GN) or alveolar hemorrhage] with induction therapy based on rituximab (RTX) or cyclophosphamide (CYC) in combination with either glucocorticoids (GC) or avacopan as part of a strategy to substantially reduce exposure to GCs (Fig. 1A) [5]. This recommendation is supported by guidelines from KDIGO [6] and national guidelines [7–11, 14]. Whereas all guidelines consider RTX to be superior to CYC in patients with relapsing disease [5, 6, 8, 10, 11, 14], RTX and CYC are generally considered to be equally effective in patients with new-onset GPA/MPA [5, 6, 8, 10, 11, 14]. In addition, KDIGO and the French guidelines recommend treating patients with severe kidney disease [defined as serum creatinine (SCr) levels >4 mg/dl/>354 μmol/L] preferentially with CYC [6, 12, 13]. KDIGO also recommends a combination of CYC and RTX [6], although there are currently no randomized clinical trials showing the superiority of this combination over RTX or CCY alone.

For patients without organ or life-threatening GPA/MPA, EULAR (Fig. 1A) [5] and UK guidelines [14] recommend initiating first-line induction therapy using RTX over methotrexate (MTX) or mycophenolate mofetil (MMF) in combination with high-dose GC or avacopan. This is because, although not proven in clinical trials, treatment with RTX potentially enables prescribers to reduce the cumulative GC dose by lowering the starting dose and intensifying the tapering schedule. However, there is limited agreement across guidelines, with Germany and France recommending first-line therapy based on RTX or MTX [8, 13], India, Japan and KDIGO recommending RTX or CYC [6, 9, 10] and the Pan American League of Associations for Rheumatology (PANLAR) and Canadian guidelines recommending MTX over RTX or CYC [7, 11].

The role of plasma exchange during induction therapy for GPA and MPA has been questioned following results from the PEXIVAS trial, which demonstrated little or no benefit for plasma exchange *vs* two different GC-based regimens on the risk of death or end-stage kidney disease in patients with severe AAV defined as an estimated glomerular filtration rate (eGFR) <50 ml/min/1.73 m^2^ or diffuse pulmonary hemorrhage [15]. However, meta-analyses of data from earlier trials suggest that patient subgroups with rapidly progressing glomerulonephritis (RPGN) may benefit from plasma exchange in terms of dialysis-independence after 1 year [16]. Consequently, most guidelines [5, 6, 8–10, 14, 17] agree that plasma exchange should be considered in addition to immunosuppression in patients with RPGN, defined as SCr >5.8 mg/dl by Indian guidelines [10], >3.4 mg/dl by KDIGO [6] and >3.4 mg/dl with or without alveolar hemorrhage and hypoxemia by EULAR, Germany, Ireland, Japan and the UK [5, 8, 9, 14, 17].

**Figure 2.**
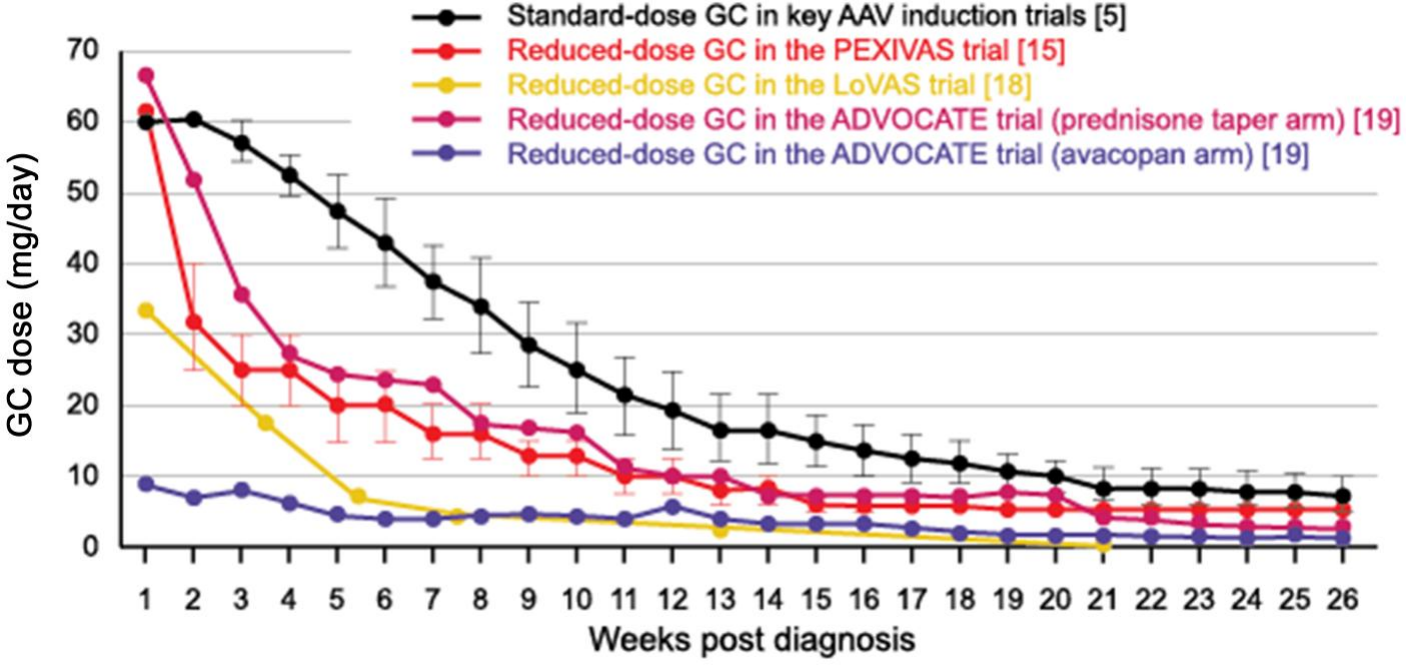
Results from PEXIVAS [15], LoVAS [18] and ADVOCATE [19] clinical trials have led to more rapid GC tapering in clinical practice. AAV, ANCA-associated vasculitis; GC, glucocortocoid. GC doses in the PEXIVAS and LoVAS trials were per protocol whereas GC doses in the ADVOCATE trial were based on actual exposure

The PEXIVAS trial [15] and the LoVAS trial [18] demonstrated similar efficacy and reduced rates of infection for a lower-dose GC regimen *vs* a standard-dose regimen. Consistent with this observation, EULAR (Fig. 1A) (and most other guidelines) recommended rapidly tapering GCs from an initial dose of 60 mg/day to 5 mg/day over a period of 4–5 months [5–11, 14, 17]. The phase 3 ADVOCATE trial [19] found that adjunctive therapy with the novel complement 5a receptor (C5aR) antagonist, avacopan, enabled substantial reductions in GC exposure in patients with GPA/MPA treated with RTX or CYC, without compromising remission or relapse rates (Fig. 2) [19]. This led AAV treatment guidelines from EULAR, Canada, Germany and Ireland to state that avacopan use may be considered as part of a strategy to reduce GC exposure in patients receiving induction therapy for GPA/MPA [5, 7, 8, 17]. Similarly, UK guidelines suggest avacopan should be considered as a steroid-sparing agent with or without a short course of GCs (tapering over 4 weeks) [14], Japanese guidelines state a preference for avacopan over high-dose GCs [9] and KDIGO guidelines suggest that avacopan should be considered as an alternative to GCs, especially in patients with an increased risk of GC toxicity and/or those with lower eGFR who may benefit from greater GFR recovery [6].

## Maintenance therapy

AAV treatment guidelines agree that patients achieving AAV remission should receive maintenance therapy with RTX or alternatively azathioprine (AZA) or MTX (leflunomide in India), while continuing their GC taper (Fig. 1B) [5–11, 13, 14]. Other than KDIGO (which recommends RTX or AZA [6]) and PANLAR (which recommends RTX in patients with severe AAV and AZA, MTX or MMF in those with non-severe disease [11]), all guidelines (including EULAR [5]) recommend RTX (500 mg every 6 months) as the standard-of-care for maintenance therapy. This recommendation was based on results from two randomized trials on maintenance therapy in patients with GPA or MPA, which showed lower relapse rates with RTX compared with AZA [20, 21]. Data from another randomized trial demonstrate that MMF is inferior to AZA for maintenance of remission [22], which itself is inferior to RTX in maintenance therapy [20, 21]. In the ADVOCATE trial, the proportion of patients maintaining AAV remission through to week 52 (the second primary end point) was greater among patients receiving avacopan (65.7%) *vs* prednisone taper (54.9%) [19]. Due to the lack of data beyond one year, AAV treatment guidelines do not recommend continuing avacopan use beyond 12 months [5, 7–9, 14]. However, this might change as long-term data emerge.

The recommended duration of RTX, AZA or MTX therapy varies between guidelines, with EULAR, UK and Canadian guidelines recommending 24–48 months [5, 7, 14] and KDIGO guidelines recommending 18–48 months [6] in patients with new-onset disease or longer (balanced against the potential risks) in those with or at increased risk of AAV relapse (Fig. 1B). In contrast, German guidelines recommend ≥36 months for conventional immunosuppressive treatment and ≥48 months for RTX [8], while Indian guidelines recommend ≥48 months [10], PANLAR recommend tailoring the duration of therapy according to patient preferences and risk factors [11], and Japanese guidelines state a preference for ‘long-term’ over ‘short-term’ therapy [9]. French guidelines recommend a total of 48 months for patients with PR3-ANCA positive AAV and initially severe and relapsing MPO-ANCA positive AAV and 24 months for newly diagnosed severe MPO-ANCA positive AAV [13].

Recommendations for reducing GC doses in people receiving maintenance therapy vary between guidelines, with EULAR and German guidelines suggesting the duration and dosage of GCs should be tailored according to the individual patient’s disease course, risk of CG-related comorbidities and patient preference [5, 8]. In contrast, PANLAR [11] and UK guidelines [14] recommend tapering GC doses for complete withdrawal. Recent preliminary results from the TAPIR trial indicate a low risk of major relapse in patients with GPA treated with RTX irrespective of whether patients received low-dose GC or no GC [23]. A post-hoc subgroup analysis of this study suggests that stopping low-dose GC in patients receiving RTX for maintenance of remission did not increase relapse rates while the benefits of low-dose GC to prevent minor relapse were only observed among patients treated with non-RTX-based regimens. Although results from the TAPIR study have currently only been reported as a congress abstract and observations are based on a post-hoc subgroup analysis with inherent methodological limitations, the data support the use of RTX as part of a first-line maintenance regimen.

## Summary and conclusions

EULAR guidelines for the induction and maintenance of remission in patients with GPA or MPA are largely in agreement with KDIGO and national guidelines, with some minor differences related to the interpretation of data and/or national health system preferences. Most guidelines agree that the introduction of RTX has improved outcomes in patients with AAV [24], and most recommend the use of avacopan as part of a strategy to substantially reduce or avoid exposure to GC [5–11, 14, 17]. Guidelines for the treatment of AAV will continue to evolve as ongoing studies provide new insights into the use of reduced-dose GC protocols, alternative (GC-sparing) treatment options, the use of plasma exchange in people with RPGN and optimal RTX-based treatment regimens.

The reviews in this supplement describe the burden of AAV [25], the role of the complement system in AAV pathophysiology [26], the benefits of complement-targeted avacopan therapy [26, 27] and key challenges in the management of GPA/MPA, based on evidence from clinical trials and real-world case studies [27].

## Data Availability

No new data were generated or analysed in support of this research.
